# Effects of Listener Position on Speech Recognition in a Simulated Multitalker Environment

**DOI:** 10.3390/audiolres16040109

**Published:** 2026-07-29

**Authors:** William J. Bologna, Courtney King, Katie Esser, Elaine V. Shaw, Karina M. Ball

**Affiliations:** Department of Speech-Language Pathology & Audiology, Towson University, 8000 York Road, Towson, MD 21252, USA; cking40@students.towson.edu (C.K.); kesser1@students.towson.edu (K.E.); eshaw7@students.towson.edu (E.V.S.);

**Keywords:** speech recognition, virtual acoustics, listener position

## Abstract

**Background/Objectives**: A listener’s physical position in a realistic environment influences the intensity of speech and noise that arrives at the two ears as well as the binaural cues available for the segregation of target signals from background noise. The purpose of this paper was to evaluate how listener position influences speech recognition in a simulated multitalker environment and to determine the extent to which strategic positioning can be leveraged to improve communication in the real world. **Methods**: Virtual acoustics were used to generate a realistic simulation of a restaurant with 12 different listener positions, which were distributed across four different tables. Speech recognition in each simulated position was measured in 64 listeners separated into two age groups (younger and older) with varying degrees of hearing sensitivity represented in both groups. **Results**: Significant differences in speech recognition were observed between tables and between seats at each table, indicating that even subtle changes in listener position can yield meaningful effects on speech recognition. The best positions tended to contain asymmetric distributions of maskers on either side of the listener, and they afforded access to spatial cues for segregation of the target from nearby maskers. Age and hearing sensitivity also contributed to speech recognition thresholds and interacted with the effects of listener position. **Conclusions**: Manipulations of listener position may be an effective way to improve communication in realistic environments. Future work is needed to develop generalizable strategies to guide patients on how to identify positions that are optimal for speech understanding in any given environment.

## 1. Introduction

One of the most common reasons patients seek audiological services is to improve their ability to understand speech in background noise. Speech-in-noise difficulty limits engagement in social environments and has been linked to social isolation, depression, and poorer overall quality of life [1,2,3]. The standard treatment approaches in audiology, such as hearing aids and cochlear implants, are designed to restore the audibility of speech for individuals with peripheral hearing loss. However, these approaches have considerable limitations in background noise [4,5]. Furthermore, many patients who report speech-in-noise difficulty have relatively normal auditory thresholds [2,6]. This limits the potential benefit they may receive from amplification, leading to considerable variability in audiological approaches to treatment [7] and patient experience with hearing healthcare [8]. There is a clinical need for new rehabilitative approaches designed to address communication challenges in noise for all patients who report this common problem.

Aural rehabilitation has been a long-standing supplement to audiological treatment [9,10,11]. One component of aural rehabilitation is to deliver practical strategies that listeners can implement to improve their communication, such as position-based recommendations for improving communication in a restaurant [12]. While these recommendations are grounded in theories of room acoustics and clinical experience, there is limited empirical evidence to support their efficacy in realistic acoustic spaces. The purpose of this paper was to begin evaluating how listener position influences speech understanding in a realistic environment. The long-term goal is to identify practical strategies that listeners could employ to improve their communication in noise as well as to provide empirical evidence to support the clinical translation of these positioning strategies as a component of aural rehabilitation.

Previous psychoacoustics research has demonstrated that spatial separation between the target talker and masker(s) is highly advantageous for speech understanding [13,14]. Spatial separation allows listeners to leverage spatially directed attention to selectively attend to the target and ignore maskers [15]. When maskers are asymmetrically positioned around the listener, spatial separation improves the signal-to-noise ratio (SNR) in the ear farthest from the noise source [14]. These two effects of spatial separation are sensitive to listener characteristics such as age and hearing sensitivity. Older adults are less able to capitalize on spatially directed attention than younger listeners [13], and hearing-impaired listeners are still limited by the reduced audibility of the target, even if the maskers are spatially separated [14]. The angle of spatial separation can accentuate the effects of age or hearing loss; smaller angles are more sensitive to age effects, whereas larger angles are more sensitive to hearing loss [16]. These studies were all conducted with rigid experimental controls imposed to test hypothesized effects of spatial separation. Their results, collectively, illustrate the inherent complexity of spatial hearing effects that can underly speech understanding in a realistic environment. Our objective in this paper was to design a more realistic spatial hearing experiment with maskers asymmetrically distributed at different angles and distances relative to the listener. In such an environment, the combined effects of spatial cues from several different maskers reflect the advantages that listeners could leverage by adjusting their position in a realistic space, such as a restaurant.

Acoustic simulations of a realistic multitalker environment can be achieved under headphones using virtual acoustics. Briefly, individual digital filters are generated for each simulated sound source at each ear, and all these signals are combined into a two-channel waveform that captures the unique binaural cues for each source, which is based on the listener’s head orientation, and the overall level for each source, which is based on the distance between the listener and each simulated source [17]. Similar approaches have been implemented in circular or spherical speaker arrays to investigate how masking effects change for maskers at different distances and angles relative to the listener [18,19]. The effects of hearing loss and hearing aid features in realistic environments have also been explored using these methods [20,21]. However, these studies typically simulate a single listener position with different masker orientations around this position. As a result, these studies provide information on how masker orientation influences speech understanding as opposed to how changes in listener position can improve communication for a particular orientation of maskers. Considering our goal of identifying practical strategies that listeners can employ, it is essential for our experimental manipulation to be one that listeners could implement in a real-world environment (i.e., moving themselves, as opposed to moving the maskers).

In this paper, virtual acoustics were used to simulate a restaurant with four tables, which each had three seats (12 listener positions). Ten competing talkers were distributed in the simulated space in probable static locations of other restaurant patrons. Each listener position afforded different SNRs at each ear, spatial cues for each source, and variable masker talker levels based on their proximity to the listener. This simulation allowed for a comparison of different listening positions within the space to determine whether clinically meaningful improvements in speech recognition could be realized by changing the listener’s position. Speech recognition was tested in a sample of listeners that varied in age and hearing status in order to capture how these listener characteristics influenced speech recognition and the effects of listener position in the virtual space. A subset of these data has been published separately to evaluate the effects of autistic traits on speech recognition in noise, and interested readers are referred there for those analyses [22]. The current analyses represent the full set of data and a comprehensive evaluation of how age, hearing sensitivity, and position influence speech recognition in a realistic multitalker environment. The long-term goal of this line of research is to establish evidence-based strategies for choosing an optimal listening position in a real-world environment. This paper represents an initial step toward that goal by documenting the magnitude of positioning effects in a simulated multitalker environment.

## 2. Materials and Methods

### 2.1. Participants

The study sample consisted of 64 native speakers of American English (40 female, 23 male, 1 non-binary) who were separated into two age groups. The younger group (*n* = 32) was aged 19–39 years (M = 24.2, SD = 4.1), and the older group (*n* = 32) was aged 51–83 years (M = 64.3, SD = 7.3). Close to half of the sample had normal audiometric thresholds (25 younger and 5 older), which are defined as thresholds ≤25 dB HL at all octave frequencies from 250 to 8000 Hz. The other participants had variations in the degree, type, and etiology of hearing loss (7 younger and 27 older). Hearing losses were generally sloping sensorineural losses in the mild-to-moderate degree range with only seven participants presenting with moderately-severe thresholds (all of which were at 8000 Hz). Three participants presented with asymmetric or unilateral losses, all of which were conductive. Hearing sensitivity was quantified in several ways for statistical modeling, and the most effective predictor of performance was the average audiometric threshold across six octave frequencies from 250–8000 Hz in the better hearing ear (see Section 3). Although hearing thresholds were associated with age, the sample included younger adults with impaired hearing alongside older adults with normal hearing to reduce the collinearity between age and hearing sensitivity in the data. The diversity in audiometric configurations, types, and degrees of hearing loss contributed to the robustness of the sample for investigating the effects of position on speech recognition as well as how those effects are influenced by age group and hearing sensitivity. Participants reported varying degrees of autistic traits, assessed with the Ritvo Autism and Asperger Diagnostic Scale–14 Screen [23], and were not selected to match any specific neurotype. The effects of autistic traits on speech recognition are beyond the scope of this paper but have been reported previously for a subset of these data from younger listeners [22]. All participants provided informed consent and were compensated in the form of course credit or gift cards, according to protocol #1494 approved by the Institutional Review Board at Towson University.

### 2.2. Virtual Restaurant

**Figure 1:**
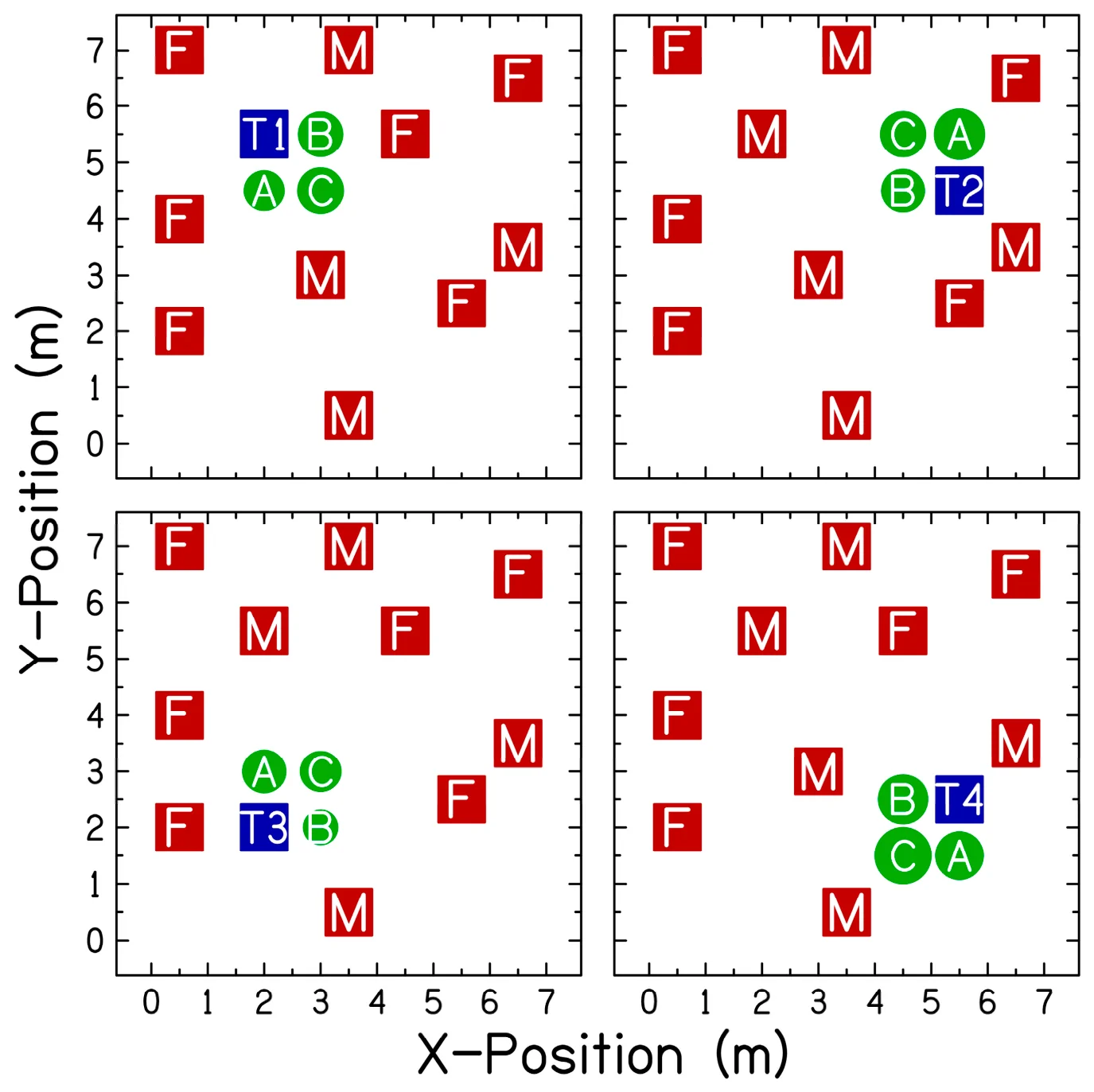
Spatial layout of targets (blue), maskers (red), and listener positions (green) across 4 tables. For maskers, M indicates a male voice, and F indicates a female voice. Target talker was always female. For positions, the size of the circle is scaled based on average performance with a larger circle indicating a lower (better) SNR at threshold. A similar figure appears in [22] and is copyrighted by ASHA, reprinted here with permission.

Figure 1 illustrates the spatial design of the virtual restaurant. The virtual space was anechoic (no simulated reverberation) with all sources positioned within a 7 × 7 m square. Each panel in Figure 1 depicts one of the four “tables” with a target talker (blue square) and three “seats” (green circles) to represent listener positions: seat A was across from the target, seat B was beside the target, and seat C was on the corner opposite the target. In all seats, the listener’s head position was simulated facing the target talker (0° azimuth) at a distance of 1 m. The target talker was a female speaker reading Bamford–Kowal–Bench sentences, which were recorded by Miller et al. [24]. Eleven masker positions were distributed unevenly in ecologically plausible positions for competing talkers in the restaurant. Seven of those masker positions were around the perimeter of the space and were consistent for all simulations of the four tables. The four remaining positions were closer to the center of the space, and only three of these maskers were included in the simulation for any given table (the fourth masker position was displaced by the target talker and 3 listener positions). Each masker position was assigned a unique narrative speech sample, all of which were recorded by Miller et al. [24] using simple prompts (describe the plot of your favorite animated film, describe your day yesterday, or provide a verbal walkthrough of your home). Eleven different male and female voices were used as maskers, and each talker was exclusively used in a single masker position in the virtual environment. This design was driven by the desire for realism, by simulating a space with a plausible arrangement of stationary competing talkers, such that the only difference between conditions was the position of the listener and target talker.

Each talker was processed to simulate spatial cues and changes in intensity with distance to the listener’s position using virtual acoustics implemented in 3D Tune-In Toolkit Binaural Test Application (3DTI; version 4.0) [25]. Prior to processing, all target and masker stimuli were root-mean-square amplitude normalized to ensure that the differences in intensity between maskers would be based on the simulated distance rather than inherent amplitude differences in the recordings. This normalization step produced an RMS amplitude consistent with 70 dB SPL at a simulated distance of 1 m. Next, each narrative was processed in 3DTI based on that masker’s position relative to each listener position. In each position, all maskers were at variable distances and angles relative to the listener’s head orientation (always facing the target talker). Processed maskers were then combined to produce a unique 10-talker masker for each position, with each masker talker individually rendered for a realistic spatial percept in azimuth and distance, which is based on the design in Figure 1. Target sentences were also processed in 3DTI but with a uniform distance of 1 m at 0° azimuth to simulate the fixed position of the target relative to the listener.

The inherent variability of masker orientations in these positions produced nominally different overall masker levels (based on the combined intensity of all competing talkers). Table 1 describes the overall masker levels in the right ear, left ear, and the average across the two ears for each position. In general, differences in the masker level between ears and across positions were less than 5 dB and reflect the natural variation that would be expected by moving through this simulated space. Output levels were confirmed through calibration with an iPad (9th generation) running the NIOSH sound level meter app (version 1.2.6.42) and a MicW iBoundary condenser microphone (Bejing, China).

### 2.3. Procedures

After obtaining consent, all participants completed a pure-tone audiogram with air and bone conduction to document their hearing status. Then, the experimental protocols were explained. Participants were shown a graphical representation of the virtual restaurant (similar to Figure 1) and told that they would be tested at each of the twelve positions. This graphical representation was available to them throughout testing, and the experimenter informed the participants which virtual seat they were in before each condition. They were instructed to repeat sentences from a female voice directly in front of them and ignore ongoing conversations around them. They were informed that the female voice would initially be loud and easy to recognize but that it would become softer as the test progressed. They were encouraged to guess when they were unsure of what they heard. The order of positions was randomized across participants, and a break was offered halfway through testing. All participants, including those with hearing loss, were tested without any ear-level amplification device. The full protocol took less than 2 h to complete.

In each position, the target intensity was adapted based on the word-level scoring of performance to efficiently sample a wide range of the psychometric functions, which is based on procedures described by Wasiuk et al. [26]. This procedure used two interleaved adaptive tracks with different criteria for level adjustment. One track decreased target level only when the participant correctly repeated all words in the sentence or made only one word error, and the other decreased target level if the participant repeated at least one correct word in the sentence. Trials from these two adaptive tracks were interleaved during testing to efficiently sample a broad area of the psychometric function. Both adaptive tracks started with a target level of 78 dB SPL and adapted in a 4 dB step size for the first two intensity changes and then reduced the step size to 2 dB for the rest of the track. Twenty-four sentences were presented in each condition, which were split evenly between the two adaptive tracks. Word-level data from both tracks were combined for estimation of the psychometric function using a general linear model.

### 2.4. Analyses

Word recognition data as a function of SNR in each position were fit to a logit function using a general linear model implemented in R (version 4.1.1) [27], using the lme4 package [28]. The average noise level across the two ears in a given position was subtracted from the target intensity level to determine the SNR for a given sentence. Across 24 sentences in each position, the SNRs were broadly distributed over a range of performance levels that facilitated estimation of the psychometric function from the logit fit. The SNR at the 50% correct point of the estimated function was extracted for each participant in each position. These thresholds were initially analyzed using repeated-measures ANOVA to determine whether position influenced the SNR required for 50% correct. Next, thresholds were analyzed using a linear mixed effects model, which included independent variables that described the listener’s position, age group, hearing sensitivity, and subject ID as a random effect. The purpose of this model was to determine the effects of age and hearing loss on performance and how those effects changed as a function of listener position. The modeling process began with a base model that only included listener position as a predictive factor along with subject as a random-effects term. Age group (younger coded as reference), various measures of hearing sensitivity, and all possible two- and three-way interactions were added to the model one at a time and tested for significance using model testing [29]. Factors that significantly improved model fit were retained, and interactions were explored with post hoc models designed to reveal the nature of the interaction.

## 3. Results

**Figure 2:**
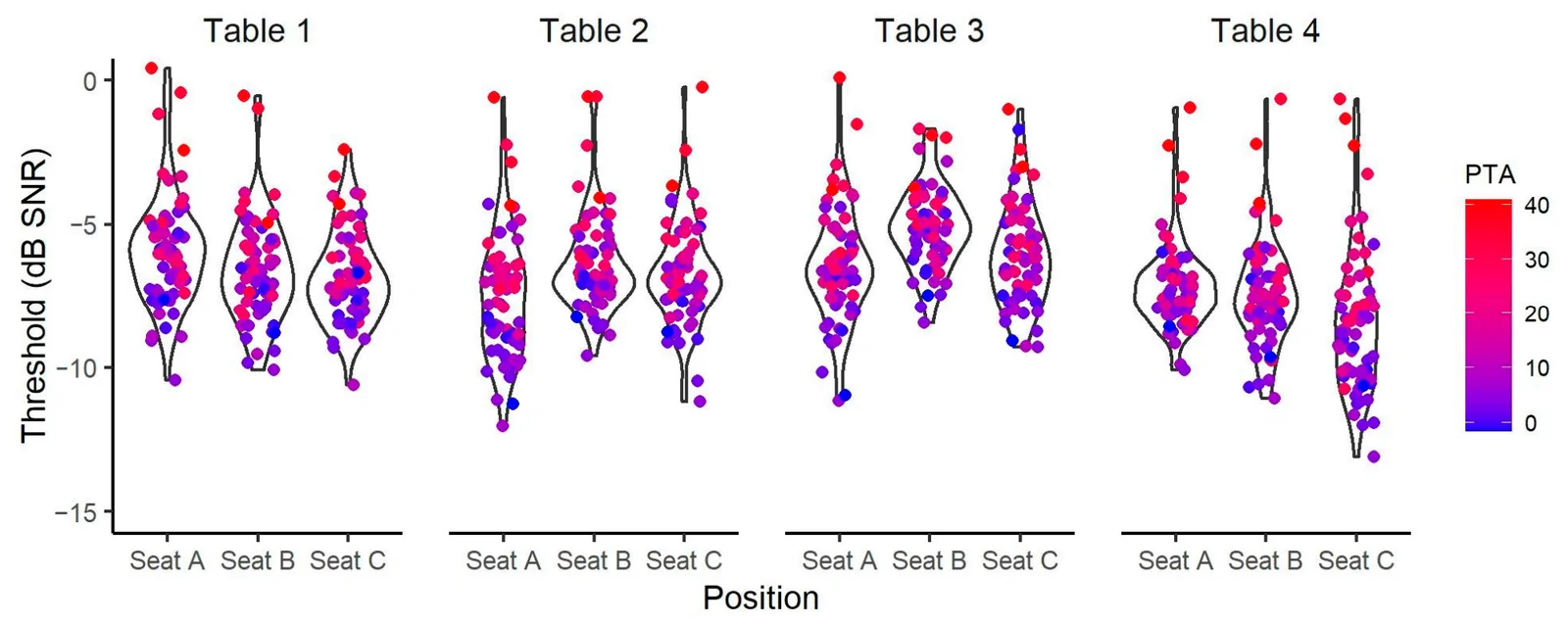
Distribution of thresholds (dB SNR) as a function of table and seat with individual data points color coded based on the better ear 6-frequency pure-tone average (warmer colors indicate more hearing loss). Significant pairwise comparisons between seats at each table are detailed below in Table 1.

The distributions of the 50% thresholds extracted from the psychometric functions in each position are displayed in Figure 2. The mean threshold in each position was also used to scale the size of the green circles in Figure 1 (larger circles indicate better performance). As a preliminary step in data analysis, these data were analyzed with a two-way repeated measures ANOVA with factors of table and seat. Results indicated significant effects of table, F(3, 189) = 113.63, *p* < 0.001, and seat, F(2, 126) = 28.04, *p* < 0.001, as well as a significant table × seat interaction, F(4.4, 276.91) = 27.83, *p* < 0.001. These effects were further explored with post hoc tests, which were all completed with Bonferroni adjustment for multiple comparisons. The significant effect of table was explored with post hoc pairwise *t*-tests, which indicated significant differences between all tables. Performance was best at Table 4, which was followed by Table 2 (Table 4 vs. 2: t(191) = −6.69, *p* < 0.001), better at Table 2 than Table 1 (Table 2 vs. 1: t(191) = −3.77, *p* < 0.01), and better at Table 1 than Table 3 (Table 1 vs. 3: t(191) = −5.34, *p* < 0.001). The significant main effect of seat was also explored with Bonferroni-adjusted post hoc *t*-tests, which indicated significant differences between all seats. Performance was generally best in seats on the corner opposite the target, followed by seats across from the target (seat C vs. A: t(255) = −2.59, *p* < 0.05), with the poorest performance in seats beside the target (seat A vs. B: t(255) = −3.91, *p* < 0.001). It should be noted that this general pattern of results associated with seat was not observed consistently at each table and should be interpreted with caution. The significant interaction between seat and table provided a more meaningful interpretation of how performance changed as a function of seat at each table. This interaction was explored in two sets of post hoc tests. First, a set of four one-way repeated measures ANOVA confirmed a significant effect of seat at each table (Table 1: F(2, 126) = 18.2, *p* < 0.001, Table 2: F(1.82, 115) = 25.9, *p* < 0.001, Table 3: F(2, 126) = 38.9, *p* < 0.001, Table 4: F(1.75, 110) = 28.7, *p* < 0.001). Next, pairwise *t*-tests were completed to evaluate the differences between seats at each table. Most of these pairwise comparisons were significant, and those results are summarized in Table 2.

Overall, these results revealed that the 12 listener positions yielded significant variance in SNR thresholds for 50% correct recognition of words in sentences. Importantly, this metric (SNR threshold) accounts for the nominal differences in noise level that existed between different positions. Thus, the results reported above describe variance in speech recognition beyond what can be explained by differences in the average noise level in each position. Based on these results, and the complex pattern of thresholds that were observed across tables and seats, further analyses using linear mixed-effects modeling (described below) used a single factor of “Position” with 12 levels rather than modeling separate effects of table and seat. A base model was constructed with only Position as a fixed effect and Subject as a random effect. The next step of analysis was to incorporate effects of age group and hearing sensitivity into the model to determine how these factors influenced threshold and interacted with the effect of listener position. The modeling process produced the following model in final form,

Threshold ~ Position × PTA × Age Group + [*Subject*], (1)

with effects therein described below. Full reporting of the model’s output can be found in Appendix A Table A1.

### 3.1. Effect of PTA

The general effect of hearing sensitivity can be visualized qualitatively in Figure 2, which shows that individual thresholds (dots) for participants with poorer hearing (warmer colors) tended to gravitate toward high SNR values compared to participants with better hearing (cooler colors). To determine how best to quantify hearing sensitivity to capture this association, several different pure-tone averages (PTAs) were computed for each participant and tested one-at-a-time in the model. These included three-frequency averages (500, 1000, and 2000 Hz), four-frequency averages (500, 1000, 2000, and 4000 Hz), and six-frequency averages (250, 500, 1000, 2000, 4000, and 8000 Hz), which were measured in the right ear, left ear, average of both ears, and in the “better hearing” ear (defined as the lower PTA between right and left ear for that participant). When added to the base model, each of these PTA measures significantly improved model fit (χ^2^ > 10.46, *p* < 0.01 in all cases). The PTA measure that improved model fit the most was the six-frequency average in the better hearing ear (χ^2^ = 41.11, *p* < 0.001). This suggests that including the full range of audiometric frequencies improved the predictive power of hearing thresholds on performance, and that effects of hearing sensitivity on performance were best described by the better hearing ear. The six-frequency average in the better ear was retained in the model, labeled as “PTA,” and used for the color coding of individual data points in Figure 2.

### 3.2. Effect of Age Group

**Figure 3:**
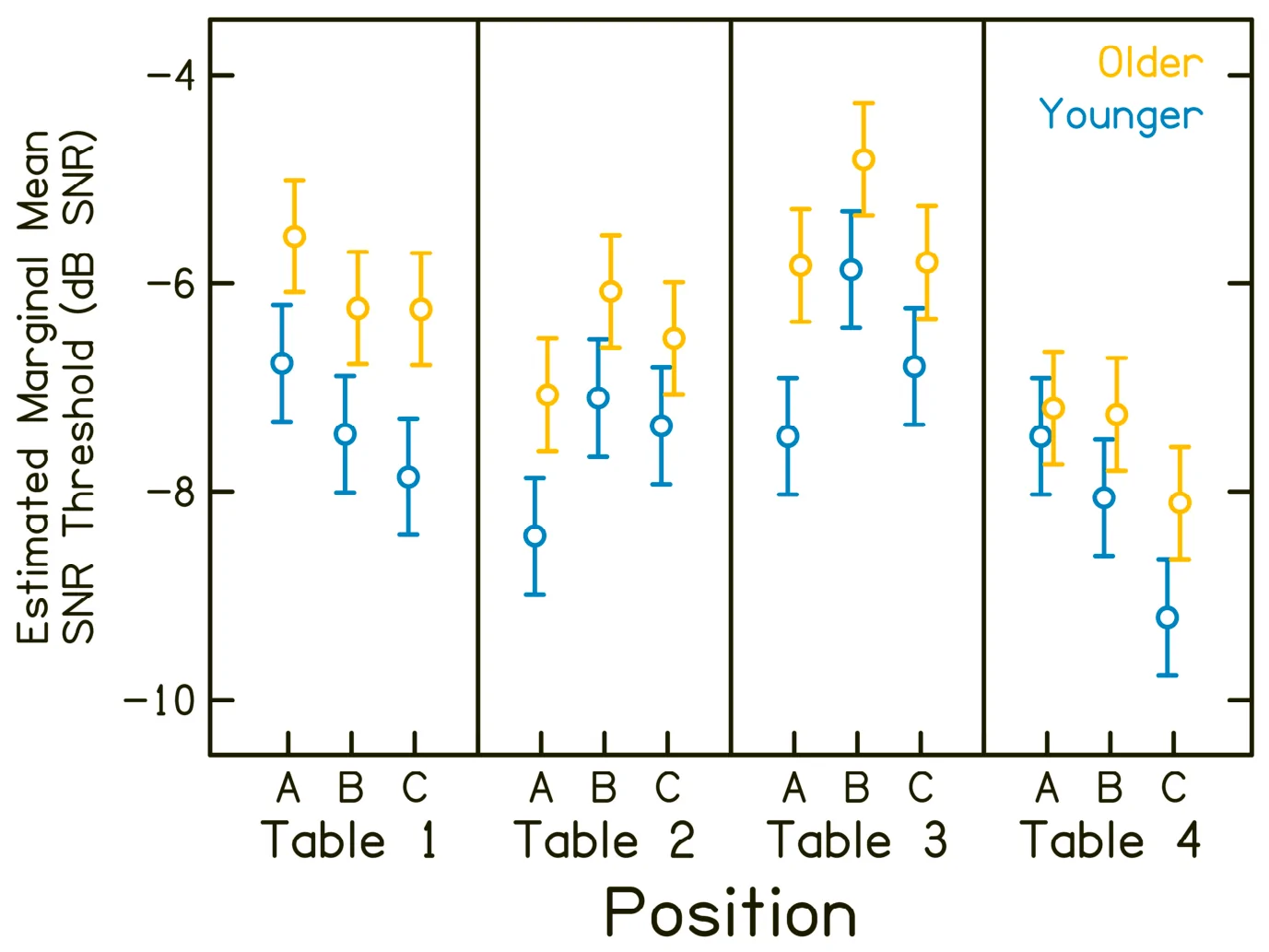
Estimated marginal means of SNR thresholds for younger adults (blue) and older adults (yellow) in each position. Error bars indicate the 95% confidence interval.

Age group also significantly improved the fit of the base model (χ^2^ = 27.79, *p* < 0.001). This effect can be visualized in Figure 3, which displays the estimated marginal means of thresholds and 95% confidence intervals for the younger group (blue) and the older groups (yellow), which were computed from the final model using the Emmeans package in R [30]. The estimated marginal means for younger adults were consistently better than those of the older adults in each position, although in some positions, the 95% confidence intervals overlap. However, an independent samples *t*-test indicated significantly greater PTA in the older group than the younger group, t(61.98) = −2.49, *p* < 0.05. Thus, the effect of age group cannot be completely disentangled from the effect of PTA. Due to the association between age group and PTA in the sample, generalized variance inflation factors (GVIFs) were calculated to assess multicollinearity [31]. Adjusted GVIFs for all factors and interactions in the final model were below 2.5, indicating an acceptable level of collinearity that is unlikely to generate problematic inflation of variance. Thus, age group was retained in the model, and interactions between significant main effects were subsequently explored.

### 3.3. Interactions

Once the main effects of PTA and age group were confirmed in the model, the two-way interaction of Age Group × PTA was tested for significance and found to significantly improve model fit (χ^2^ = 6.21, *p* < 0.05). To interpret this interaction, two post hoc models were generated by splitting the data by age group. A separate model was constructed for each age group to determine the effect of PTA on performance among the younger and older participants. Results indicated that the effect of PTA on threshold was stronger among older participants (*ß* = 0.11) than it was among younger participants (*ß* = 0.04). This finding is consistent with the idea that age-related changes beyond audibility, such as reduced temporal processing and cognitive decline, make it harder to compensate for reduced audibility due to hearing loss [32,33,34].

Additional two-way interactions were observed between Position × Age Group (χ^2^ = 43.76, *p* < 0.001) and Position × PTA (χ^2^ = 69.86, *p* < 0.001). The nature of these interactions suggested that the effects of age group and PTA differed depending on the listener’s position. The pattern of the interaction with age group can be visualized in Figure 3: some positions produced marginal means that differed between groups (non-overlapping confidence intervals), including all seats at Table 1, Table 2 seat A, and Table 3 seat A. Other positions produced marginal means with overlapping confidence intervals, indicating minimal differences between age groups (Table 4 seats A and B). The pattern of the interaction with PTA across positions was qualitatively similar to that of age group, which was due to the inherent association between age and PTA in the sample. A median split of the entire sample based on PTA (rather than age group) produced very similar subgroups of participants to those displayed in Figure 3. Thus, these data indicate that the effects of position differed for younger and older adults with varying degrees of hearing loss but cannot fully disentangle which positions were most sensitive to aging effects vs. effects of hearing loss.

Finally, the three-way interaction of Position × PTA × Age was tested for significance but did not improve the fit of the model (χ^2^ = 11.97, *p* = 0.37). Thus, the three-way interaction was removed, leaving a final model with main effects of position, PTA, age group, all their associated two-way interactions, and subject as a random effect. The complete modeling output can be found in Appendix A Table A1.

## 4. Discussion

Listener position in realistic environment influences the overall SNR at each ear, the relative intensity of individual masker voices in the background, and the availability of spatial cues for segregation of the target from maskers. Each of these factors is known to produce measurable effects of speech recognition in laboratory experiments that have investigated them in isolation [18,19,35,36]. Here, these factors were allowed to covary naturally with listener position in a simulated environment, which was generated under headphones using virtual acoustics. Results indicated that the combination of these psychophysical effects produce meaningful changes in speech recognition across listener positions even when position changes are as subtle as a 1 m change in seat at a given table. On average, SNR thresholds improved by 3.6 dB when the listener moved from the least advantageous to the most advantageous position. Notably, this crosses the threshold for a meaningful improvement in SNR, as rated by listeners with and without hearing loss [37]. This suggests that patients may be willing and able to leverage strategic positioning decisions to improve their communication in a real-world restaurant environment. Realizing the potential utility of these effects for patient use will require additional research to identify generalizable strategies that are effective across a wide range of environments. The current data represent the first step toward that goal.

In these data, speech recognition performance was best at table four. This table had the lowest overall noise level but differed from other tables by only 1–2 dB. Note also that performance in these data was described in terms of SNR thresholds, which was calculated as the target level at 50% correct minus average noise level between the ears in that position. As such, the nominal 1–2 dB difference in noise level between positions was already accounted for in calculation of threshold. For this reason, it is unlikely that the advantage of listening at table four was driven by small differences in the overall noise level. Table four also had the most asymmetric distribution of noise sources with lower noise levels in the right ear (opposite most of the maskers in the room). It is likely that listeners leveraged the right ear for better-ear listening at table four, as seen previously in studies with asymmetric masker configurations [35]. In terms of tractable listening strategies for real-world environments, patients should prioritize quieter tables, particularly positions in which most of the noise sources are asymmetrically distributed to one side.

The effects of spatial cues in these data are complex, as all positions were generated for the spatial perception of maskers at different discrete locations. There were no “colocated” maskers, as in most laboratory studies of spatial release from masking [13,36]. However, positions did afford different amounts of azimuthal separation of target and maskers. For example, when the listener was at table three seat B, they were facing a female target talker that had a female masker 1.5 m behind them (both at 0° azimuth for the listener). The similarity of voice features or gender between a target and masker is known to induce informational masking effects [38], and this effect likely contributed to the difficulty of speech recognition in this position. Speech recognition in seat B was significantly poorer than in seats A and C at table three, and this effect may reflect the differences in the spatial orientation of maskers across seats. In seats A and C, spatial cues would be an effective means of segregating the target from this female masker, whereas this cue would not be as effective in seat B. Although the simulated environment in this experiment was considerably more complex and less controlled than a typical laboratory spatial hearing task, recognizable patterns of results were observed across positions consistent with theories of spatial release from masking. Thus, patients will be able leverage spatial cues most effectively when competing talkers are not directly behind their communication partner.

This paper adds to a growing body of literature on physical listening behaviors that can improve auditory performance in realistic noisy environments. Another example of beneficial listener behavior is head movements, which can support localization abilities and speech recognition in complex environments [39,40]. Continued work in this area builds evidence for new rehabilitative therapeutic approaches in audiology that are focused on physical listening behaviors and strategic movements to support communication in noise. Importantly, these strategies are appropriate for all individuals who report difficulty in noise regardless of their audiometric profile. As such, these new rehabilitative practices would fill a known gap in clinical practice: treatment of individuals with speech-in-noise complaints despite relatively normal audiometric thresholds [8].

Several limitations of the study’s design were noted, and future work is underway to improve the methodology in this line of research. First, these data are based on a single masker orientation which produced different combinations of masking effects across positions. It is unclear from these data how listeners should weigh different positioning strategies when choosing a particular seat or table (e.g., choosing between lower noise level or better spatial separation of noise sources). As such, the strategies identified from these data should be interpreted with caution, as they may not generalize to all listening environments. Future studies are underway to address this limitation by generating a large set of realistic masker orientations based on photographs of people gathering in real-world environments. In future work, several listener positions will be tested with a large and diverse set of masker orientations. This necessary expansion of data collection will support the development of generalizable strategies that patients could use in any complex listening environment.

The acoustic simulation itself also has notable limitations. These stimuli were generated without any simulated reverberation, which is not characteristic of a real-world space. Recent work has shown that reverberant energy can alter patterns of masking and the extent to which spatial separation improves speech recognition thresholds [41]. Future simulations will incorporate reverberant energy to account for these effects and improve the realism of the stimuli. The virtual simulation of binaural cues under headphones could also be considered a limitation due to the reliance on non-individualized head-related transfer functions that may not align with all listeners’ real-world experience. Future work will be completed in a sound field with a circular speaker array, allowing for binaural cues to be realized naturally by all listeners based on their own head and ear anatomy. This transition to sound-field testing will allow for future investigations to analyze head turns and other subtle behavioral strategies that listeners may employ during difficult listening tasks. Finally, the masker narrative recordings used here were all recorded with the microphone at 0° azimuth. This effectively simulates a masker who is facing the listener regardless of the listener’s head orientation. Recent work has demonstrated the effect of masker head orientation on speech recognition in simulated multitalker environments [42]. Future work will utilize off-center-microphone recordings of masker narratives [24] in order to produce more ecologically valid simulations with variations in masker head orientation. These iterative improvements in the simulation design will improve the generalizability of future results to real-world environments.

Finally, the extent to which these data can fully disentangle the effects of age and hearing sensitivity on speech recognition in different positions is limited. The covariance of age and PTA in this sample is reflective of general population demographics, but it creates a difficult confound between these participant-level factors [33]. It is difficult to assess with certainty whether the effects and interactions observed here are more reflective of age effects or effects of peripheral pathology. Our modeling approach was simply to incorporate both factors into the model independently in order to capture the variance in performance that is expected to occur with age and PTA. It should be noted, however, that the benefits derived from different positioning strategies are likely to vary based on the age and hearing sensitivity of the patient. In these data, some positions produced fairly similar thresholds for younger and older adults, whereas other positions produced much larger differences between groups. Due to the covariance of age and PTA in the sample, this observed effect in the data cannot be cleanly interpreted as an age effect. In a parallel analysis, the sample was split based on the median PTA, which resulted in essentially the same split of the dataset, and the outcome of the analysis was the same. Therefore, the conservative conclusion is that either age or PTA (or their combined effects) is likely to influence which positioning strategy would be optimal for a given listener. Future work is needed to fully explain how these listener-specific factors (age and hearing sensitivity) interact with position-specific factors that influence speech understanding in realistic environments.

## 5. Conclusions

The masking effects that listeners experience in a complex environment depend on the position and orientation of maskers relative to the listener and their communication partner. By manipulating their own position, listeners have the agency to minimize these masking effects and improve their speech recognition. In this paper, average SNR thresholds improved by 3.6 dB when the listener moved from the least advantageous to the most advantageous position. Based on these initial results, positions with asymmetric masker orientations and good access to spatial cues should be favored over positions with more symmetrical orientations or poorer access to spatial cues. Considerably more research is needed to fully describe the general principles for optimal positioning in a complex listening environment. This represents an important future direction for the audiological rehabilitation of patients who report difficulty in background noise even when they present with normal auditory thresholds.

## Figures and Tables

**Table 1:** Masker levels in the left ear, right ear, and average across the two ears, displayed as a function of listener position. Copyrighted by ASHA [22]. Reprinted with permission.

Position	Left Ear (dB SPL)	Right Ear (dB SPL)	Average (dB SPL)
Table 1—Seat A	73.1	73.4	73.2
Table 1—Seat B	73.2	74.7	73.9
Table 1—Seat C	74.0	74.3	74.2
Table 2—Seat A	73.1	72.5	72.8
Table 2—Seat B	72.1	73.3	72.7
Table 2—Seat C	71.8	73.0	72.4
Table 3—Seat A	72.3	73.0	72.6
Table 3—Seat B	72.2	72.4	72.3
Table 3—Seat C	71.9	72.5	72.2
Table 4—Seat A	71.8	69.3	70.6
Table 4—Seat B	73.3	71.3	72.3
Table 4—Seat C	73.3	70.4	71.9

**Table 2:** Results of post hoc pairwise comparisons between seats at each table.

Table	Seat Contrast	Test Statistic	Bonferroni-Adjusted *p* Value
Table 1	A vs. B	*t*(63) = 5.01	*p* < 0.001 ***
	A vs. C	*t*(63) = 5.79	*p* < 0.001 ***
	B vs. C	*t*(63) = 1.22	*p* = 0.68
Table 2	A vs. B	*t*(63) = −7.37	*p* < 0.001 ***
	A vs. C	*t*(63) = −4.24	*p* < 0.001 ***
	B vs. C	*t*(63) = 2.47	*p* < 0.05 *
Table 3	A vs. B	*t*(63) = −8.10	*p* < 0.001 ***
	A vs. C	*t*(63) = −2.35	*p* = 0.07
	B vs. C	*t*(63) = 6.42	*p* < 0.001 ***
Table 4	A vs. B	*t*(63) = 2.22	*p* = 0.09
	A vs. C	*t*(63) = 6.37	*p* < 0.001 ***
	B vs. C	*t*(63) = 5.36	*p* < 0.001 ***

* Indicates significance at alpha level of 0.05. *** Indicates significance at alpha level of 0.001.

## Data Availability

Primary data are openly available on our OSF page: https://osf.io/exvf3.

## Abbreviations

The following abbreviations are used in this manuscript:

SNR	Signal-to-noise ratio
PTA	Pure tone average
GVIF	Generalized error inflation factor

## Appendix A

**Table A1:** Linear mixed-effects model output for Threshold ~ Position × PTA × Age Group + [*Subject*]. For fixed effects of position and interactions with position, Table 1—Seat A was used as the reference. For age group, the younger adult group was used as the reference.

Fixed Effects	Estimate	Standard Error	*t* Value
(Intercept)	−7.29	0.30	−23.95
Age Group	0.27	0.54	0.49
PTA	0.04	0.03	1.57
Table 1—Seat B	−0.47	0.26	−1.85
Table 1—Seat C	−0.69	0.26	−2.69
Table 2—Seat A	−1.88	0.26	−7.33
Table 2—Seat B	−0.21	0.26	−0.82
Table 2—Seat C	−0.53	0.26	−2.06
Table 3—Seat A	−0.59	0.26	−2.32
Table 3—Seat B	1.47	0.26	5.75
Table 3—Seat C	0.26	0.26	1.00
Table 4—Seat A	−0.59	0.26	−2.29
Table 4—Seat B	−1.36	0.26	−5.31
Table 4—Seat C	−3.17	0.26	−12.39
Age Group × PTA	0.07	0.03	2.47
Age Group × Table 1—Seat B	−0.01	0.38	−0.04
Age Group × Table 1—Seat C	0.39	0.38	1.03
Age Group × Table 2—Seat A	0.13	0.38	0.36
Age Group × Table 2—Seat B	−0.19	0.38	−0.51
Age Group × Table 2—Seat C	−0.38	0.38	−1.00
Age Group × Table 3—Seat A	0.41	0.38	1.10
Age Group × Table 3—Seat B	−0.16	0.38	−0.43
Age Group × Table 3—Seat C	−0.23	0.38	−0.61
Age Group × Table 4—Seat A	−0.95	0.38	−2.52
Age Group × Table 4—Seat B	−0.42	0.38	−1.12
Age Group × Table 4—Seat C	−0.12	0.38	−0.33
PTA × Table 1—Seat B	−0.02	0.02	−0.92
PTA × Table 1—Seat C	−0.03	0.02	−1.79
PTA × Table 2—Seat A	0.02	0.02	0.97
PTA × Table 2—Seat B	−0.01	0.02	−0.56
PTA × Table 2—Seat C	−0.01	0.02	−0.34
PTA × Table 3—Seat A	−0.01	0.02	−0.48
PTA × Table 3—Seat B	−0.04	0.02	−2.57
PTA × Table 3—Seat C	−0.02	0.02	−1.27
PTA × Table 4—Seat A	−0.01	0.02	−0.54
PTA × Table 4—Seat B	0.01	0.02	0.29
PTA × Table 4—Seat C	0.06	0.02	3.26
